# Limb-sparing reconstruction using extracorporeal irradiation and autograft recycling for primary malignant bone tumors

**DOI:** 10.1016/j.ctro.2026.101189

**Published:** 2026-05-19

**Authors:** Edona Dreshaj, Nicolas Danthez, Fernanda G. Herrera, David Patin, Maud Marguet, Antonia Digklia, Ana Dolcan, Giovanni Dei Tos, Gilles Udin, Stéphane Cherix, Rémy Kinj

**Affiliations:** a Faculty of Biology and Medicine, University of Lausanne, Lausanne, Switzerland; b Department of Oncology, Radio-Oncology Service, Lausanne University Hospital, Switzerland; c Institute of Radiation Physics, Lausanne University Hospital, Switzerland; d Department of Orthopedics and Traumatology, Lausanne University Hospital, Switzerland

**Keywords:** Extracorporeal irradiation, Autograft recycling, Bone sarcoma, Chondrosarcoma, Ewing sarcoma

## Abstract

**Introduction:**

Autograft recycling (AR) using extracorporeal irradiation (ECI) enables limb-sparing reconstruction following resection in patients with primary malignant bone tumors (MBTs). We evaluated the oncological and functional outcomes after this procedure in a single-institution cohort.

**Materials and methods:**

This retrospective cohort study included patients with MBTs treated between December 2016 and February 2024 with en-bloc resection, AR using a single fraction of 50 Gy ECI, and bone reimplantation.

**Results:**

Twenty patients were included, with a median age of 38 years (min–max range, 18–80). The predominant histological subtypes were chondrosarcoma (N = 10), osteosarcoma (N = 4), and Ewing’s sarcoma (N = 3). The pelvis was the most frequently treated site (N = 11), followed by the upper limbs (N = 5) and the lower limbs (N = 3). All patients underwent en-bloc resection followed by a single fraction of 50 Gy ECI and reimplantation of the bone. Median follow-up was 17.1 months (min–max range, 2.5–80.6). The 2 years overall survival (OS) rate was 94.4% (Cl 95%, 83.8–100). Local control at 1 year and 2 years was 100% (Cl 95%, 100–100), and 62.5% (Cl 95%, 29.1–96.1). Functional outcomes were graded as good to excellent in 12 patients (60%) and fair to poor for 8 patients (40%). In multivariate analysis, a larger tumor size was significantly associated with local relapse (p = 0.02), while re-interventions were negatively associated with OS (p = 0.026).

**Conclusion:**

AR using ECI achieved encouraging oncological and functional outcomes in patients with MBTs and represents a valuable reconstructive technique in limb-sparing surgery.

## Highlights

• AR with ECI is used for limb-sparing reconstruction after resection of malignant bone tumors.
• The technique provides encouraging oncological outcomes with good local disease control.
• Larger tumor size is associated with an increased risk of local relapse, while re-interventions negatively impact OS.
• AR with ECI represents a valuable reconstructive option in limb-sparing surgery.

## Introduction

Primary malignant bone tumors (MBTs), including osteosarcoma, chondrosarcoma, and Ewing’s sarcoma arise directly from bone tissue [1]. Primary MBTs are rare entities, accounting for approximately 0.2% of all malignant tumors [2], [3]. Advances in systemic therapy, and radiotherapy, and reconstructive surgery have significantly transformed the management of bone sarcomas. These developments have enabled limb sparing approaches to become standard, relegating amputation to selected cases rather than routine practice [4], [5]. Autograft recycling (AR) is a limb-preserving technique that involves en-bloc resection of the tumor-bearing bone, extracorporeal irradiation (ECI) of the specimen with a single ablative dose of radiotherapy, followed by reimplantation of the sterilized bone segment in its original anatomical position for reconstructive surgery. Beyond preserving limb function, this strategy offers several advantages. The procedure provides multiple advantages. The recycled autograph precisely matches the dimensions of the resected segment, obviates the need of bone banking, and avoids complications associated with allografts, including anatomical mismatch, limited availability, pathogen transmission, and increased costs [6], [7], [8]. Furthermore, irradiating the bone outside of the body offers the possibility to target the tumor-bearing bone with a high dose of radiation while sparing *peri*-tumoral structures from unintended exposure to radiation [9]. Several studies have reported favorable local control (LC) and disease-free survival (DFS) rates following limb-sparing surgery with ECI in the management of bone sarcoma [6], [7], [10]. In this study, we aimed to evaluate the outcome of patients with MBTs treated within our institution with this approach, focusing on the oncological and functional outcomes.

## Patients and methods

Between December 2016 and February 2024, 20 consecutive patients with MBTs underwent en-bloc tumor resection, followed by ECI and reimplantation (autograft recycling) at Lausanne University Hospital (CHUV). Patient management was coordinated through a multidisciplinary sarcoma tumor board to ensure optimal diagnostic and therapeutic decision-making. Baseline evaluation included detailed medical history and comprehensive physical examination, and magnetic resonance imaging (MRI) to define the local tumor extent. Histopathological diagnosis was established on biopsy specimens. Staging was performed using positron emission tomography (PET) or thoracoabdominal computed tomography (CT). Functional status was assessed pre-operatively based on patient’s daily activities using ECOG performance scale [11]. The study was approved by the local ethics committee (CER-VD: 2024–01221).

### Surgery and extracorporeal irradiation (ECI)

**Fig. 1:**
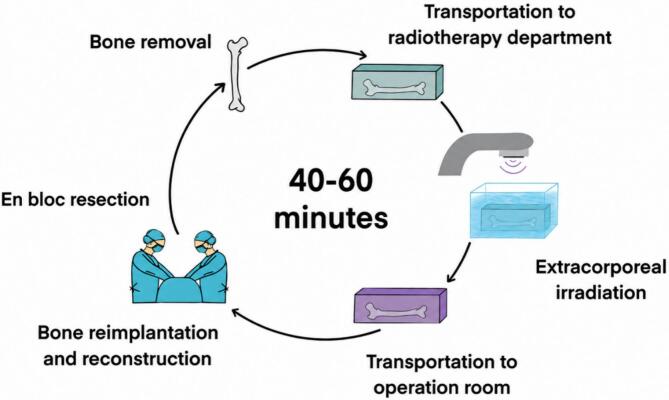
Illustration of en-bloc resection and extracorporeal irradiation.

In the operating room, en bloc oncologic resection of the tumor-bearing bone was performed. Surrounding soft tissues were meticulously removed and submitted for pathology margin evaluation. To achieve optimal radiation dose distribution, each bone was sealed in a water-filled, hermetically closed container wrapped in sterile drapes and placed on sterile plastic bags for transfer to the radiotherapy room. In the radiation department, bone segments were centered in a cylindrical, water-filled canister and stabilized between two tissue-equivalent (solid water) plates. The assembly was secured to the base of the water tank with hook-and-loop fasteners to prevent buoyancy-related movement. Specimens underwent 3D irradiation using parallel opposed fields (90° and 270°) from a linear accelerator with 10-MV photon beams, delivering a single fraction dose of 50 Gy to each bone [6], [7]. The full process, including surrounding soft tissue removal, transportation and irradiation, takes about 40–60 min. Meanwhile, frozen-section biopsies from osteotomy sites were obtained to confirm margin status and the operating room was prepared for reimplantation (redrapping, hemostatis control and osteosynthesis preparation). Following irradiation, the autograft was returned to the operating room, removed from sterile packaging and reimplanted in its original anatomical position. Before re-implantation, whenever possible, the residual tumor was curated from the irradiated bone, and the defect was filled with antibiotic loaded cement, to enhance its mechanical strength. It was then fixed using implants and joint prostheses commonly used in the traumatology and orthopedics department. (Fig. 1). An additional bone graft (vascularized fibula) could be used for reconstruction. A procedural video recorded at CHUV is available online [12].

### Follow-up

After the three-steps procedure en-bloc resection, ECI, and reimplantation— patients underwent post-operative physiotherapy for approximately 3–6 months. Surveillance imaging was tailored according to tumor grade. Patients with high-grade tumors were monitored every 3 months using thoracoabdominal CT and local MRI (1.5 Tesla), whereas those with low-grade tumors were evaluated every 6 months.

Functional outcomes were graded using a four-category functional scale [7], [13]. Postoperative complications were classified according to the Common Terminology Criteria for Adverse Events (CTCAE) [14].

### Chemotherapy

Patients received neoadjuvant chemotherapy and/or adjuvant systemic therapy and/or radiotherapy according to their specific diagnosis. Details of systemic treatments are described below.

### Statistical analysis

Statistical analyses were performed using JMP® version 17 (SAS Institute, Cary, NC, USA). Survival endpoints were evaluated using the Kaplan–Meier method, and group comparisons were conducted using the Log-rank test for censored data [15], [16]. A two-sided p-value of < 0.05 was considered statistically significant. Confidence intervals (CIs) were derived from standard errors. For quantitative variables, cut-off values were determined based on the median. To identify factors potentially associated with outcomes, Log-rank tests were first applied. Variables with a p-value ≤ 0.15 in the univariate analysis were subsequently included in a multivariate analysis using the Cox proportional hazards regression model.

## Results

### Patients

Twenty patients (13 men and 7 women) were included, with a median age at diagnosis of 38 years (min–max range: 18–80). The predominant histologies were chondrosarcoma (N = 10, 50%), osteosarcoma (N = 4, 20%), and Ewing’s sarcoma (N = 3, 15%). Tumors were more frequently located in the pelvis (n = 11), followed by the lower limbs (n = 5) and upper limbs (n = 3). Median tumor size was 8 cm (min–max range, 4–44 cm).

Nineteen patients presented with localized disease (95%), while one patient (5%) had metastatic disease at diagnosis. The type of resection, the reconstruction method and the need for additional bone graft are described in Table 1. At baseline, ECOG performance status was 0 in 10 patients, and 1 in 10 patients.

**Table 1:** Patient demographics and treatment characteristics.

**Demographics**	N = 20 (%)
**Median Age**, years (range; min–max)	38 (18–80)
**Sex**	
Male	13 (65%)
Female	7 (35%)
**Diagnosis**	
Chondrosarcoma	10 (50%)
Ewing	3 (15%)
Osteosarcoma	4 (20%)
Other	3 (15%)
**ECOG Performance Status**	
0	10 (50%)
1	10 (50%)
**Location**	
Pelvis	11 (55%)
Lower limb	5 (25%)
Upper limb	3 (15%)
Other	1 (5%)
**Tumor grade**	
G1	7 (35%)
G2	6 (30%)
G3	7 (35%)
**Stage**	
IIB	1 (5%)
IA	2 (10%)
IB	3 (15%)
IIA	5 (25%)
IIB	4 (20%)
III	4 (20%)
IVA	1 (5%)
**Treatment characteristics**	
**Neo-adjuvant treatment**	
Yes	6 (30%)
No	14 (70%)
**Neoadjuvant treatment type** (N = 6)	
Chemotherapy	6 (100%)
Other	0 (0%)
**Median time from chemotherapy to surgery** (days, (range; min–max))	27 (16–37)
**Median tumor size** (cm, min–max)	8 (4–44)
**Surgical Margin**	
R0	14 (70%)
R1	5 (25%)
R2	1 (5%)
**Type of resection**	
Segmental non articular	6 (30%)
Partial articular	8 (40%)
Complete joint resection	6 (30%)
**Reconstruction type**	
Plate and/or nail fixation	14 (70%)
Total joint prothesis and plate fixation	6 (30%)
**Additional bone graft**	
No	18 (90%)
Yes (vascularized fibula graft)	2 (10%)

### Treatment characteristics

Six patients (30%) received neoadjuvant chemotherapy. Of these, three patients with Ewing sarcoma were treated according to the Euro-Ewing 2012 protocol, which consisted of alternating intravenous cycles of vincristine, doxorubicin, and cyclophosphamide (VDC) and ifosfamide and etoposide (IE)[17]. One patient with high-grade osteosarcoma received neoadjuvant chemotherapy according to the EURAMOS-1 protocol, including methotrexate, doxorubicin, and cisplatin (MAP)[18]. Two other patients received individualized neoadjuvant chemotherapy based on histopathological findings (MAP, and doxorubicin, cisplatin, ifosfamide, methotrexate association according to EUROBOSS). Surgery was performed at a mean interval of 27 days (min–max range, 16–37) after completion of the final neoadjuvant chemotherapy cycle. Post-resection, clear margins (R0) were achieved in 14 patients (70%), while R1 margins were observed in five patients (25%) and R2 in one patient (5%) (Table 1). Among the five patients with R1 margins, three had grade 1 tumors and two had grade 2 tumors. The single R2 resection involved a grade 1 chondrosarcoma.

Postoperatively, four patients (20%) received adjuvant treatment: three received chemotherapy (75%) and two received radiotherapy (50%). Of the three patients receiving chemotherapy, two had Ewing sarcoma treated according to the Euro-Ewing 2012 protocol, and one received adjuvant chemotherapy based on clinical decision-making. Radiotherapy was administered to one patient due to marginal surgical resection, and to a second patient as pulmonary dissemination prophylaxis in Ewing sarcoma, alongside with chemotherapy (Table 2).

**Table 2:** Patients and lesions outcomes.

**Patients outcome**	N = 20 (%)
**Median Follow-up** (months, range min–max)	17.1 (2.5–80.6)
**Adjuvant treatment**	
Yes	4 (20%)
No	16 (80%)
**Adjuvant treatment type (N = 4)**	
Chemotherapy	3 (75%)
Radiotherapy	2 (50%)
**Overall survival**	
At 1 year	94.4% (83.8–100)
At 2 years	94.4% (83.8–100)
**Local control**	
At 1 year	100% (100–100)
At 2 years	62.5% (29.1–96.1)
**Disease free-survival**	
At 1 year	94.4% (83.8–100)
At 2 years	47.2% (15.1–79.1)
**Metastatic free-survival**	
At 1 year	94.4% (83.8–100)
At 2 years	82.6% (59.1–100)
**Treatment of relapse (N = 4)**	
Chemotherapy	4 (100%)
Surgery	3 (75%)
Radiotherapy	2 (50%)
Cryoablation	1 (25%)
**Acute post-surgery complication**	
G0/G1	6 (30%)
G2	5 (25%)
G3	9 (45%)
G4/5	0 (0%)
**Post-surgery complication type**	
Orthopedic/stability	6 (30%)
Neurological Pain	4 (20%)
Other	4 (20%)
**Surgical re-intervention**	
Yes	12 (60%)
No	8 (40%)
**Median number of surgical re-interventions** (N = 12, range min–max)	3 (1–12)
**Complete bone union**	
Yes	18 (90%)
No	2 (10%)
**Median time to full bone union (months, min max range)**	8.5 (3.0–25.2)
**Functional outcome scale**	
Excellent	4 (20%)
Good	8 (40%)
Fair	4 (20%)
Failure	4 (20%)

### Oncologic and functional results

Median follow-up was 17.1 months (min–max range: 2.5–80.6). Two-year OS rate was 94.4% (95% Cl, 83.8–100). Five patients experienced disease recurrence: three local and two metastatic relapses, both involving lung metastases. LC was 100% at 1 year and 62.5%(Cl 95%, 29.1–96.1) at 2 years. Metastasis-free-survival at 1 and 2 years was 94.4% (95% Cl, 83.8–100) and 82.6% (95% Cl, 59.1–100), respectively. DFS was 94.4% (95% Cl, 83.8–100) at 1 year and 47.2% (95% Cl, 15.1–79.1) at 2 years (Table 2).

### Postoperative complications and functional outcomes

Complete bone union was achieved in 80% of patients, with a median time to complet bone union of 8.5 months (min–max range: 3.0–25.2) (Table 2).

Six patients (30%) experienced no or only minor complications (CTCAE G1), five (25%) had G2 complications, and nine patients (45%) developped G3 complications; no grade 4 or 5 events occurred.

Orthopedic complications were the most frequent (n = 6, 30%), including prosthetic dislocation, implant loosening, and hardware-related discomfort. Neuropathic pain occurred in 4 patients (20%). Overall, 12 patients (60%) required surgical re-interventions, with a median number of three procedures (min–max range, 1–12). None of the re-interventions was needed to favor bone union.

**Fig. 2:**
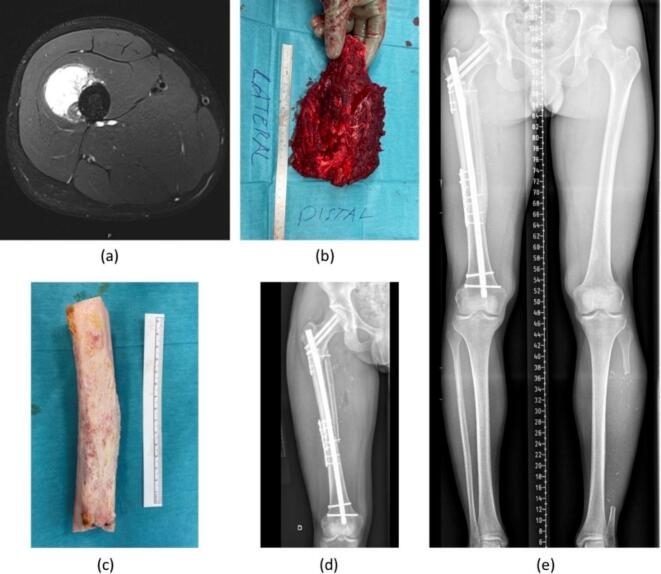
MRI scan showing periosteal osteosarcoma of the right femur in a 18-year-old male patient (a). En-bloc resection of the tumor bearing bone (b). After tissue removal, the bone was irradiated with 50 Gy using ECI (c). Re-implantation of the irradiated bone with orthopaedic fixation devices and a vascularized graft using the contralateral fibula (d). Bone union at the osteotomy sites 8 months after surgery (e).

Functional outcomes were classified as good to excellent in 12 patients (60%) and fair to poor in 8 patients (40%). An illustrative case is presented in Fig. 2**.**

### Prognosis factors analysis

On univariate and multivariate analyses, a larger tumor size > 8 cm was significantly associated with inferior LC (p = 0.04 and p = 0.02, respectively).

Univariate analysis also revealed that patients requiring surgical re-interventions after en-bloc surgery had worse overall survival (p = 0.05) compared to those undergoing a single procedure and was confirmed as an independent prognostic factor after multivariate analysis (p = 0.026). Other factors analyzed did not significantly impact on outcomes (Table 3).

**Table 3:** Prognosis factors for Local Control and Overall Survival Rate at 24 months.

**Variable**	**Local Control**	**Log rank P-value/ Cox-regression P-value**	**Overall survival**	**Log rank P-value/ Cox-regression**
**Age**<38 y-o (N = 10)≥38 y-o (N = 10)	80.0% (45–100) 66.7% (13.3–100)	p = 0.21	100% (100–100) 88.9% (68.3–100)	p = 0.51
**Gender**Female (N = 7)Male (N = 13)	100% (100–100) 60% (17–100)	p = 0.13/NS	85.7% (59.8–100) 100% (100–100)	p = 0.92
**ECOG**0 (N = 10)1 (N = 10)	75% (32.5–100)50% (1–99)	p = 0.62	100% (100–100) 90% (71.4–100)	p = 0.053/NS
**Diagnostic**Chondrosarco. (N = 10)Other (N = 10)	66.7% (29.7–100)50.0% (0–100)	p = 0.68	100% (100–100) 88.9% (68.3–100)	p = 0.56
**Grade**Low (N = 7)High (N = 13)	100% (100–100) 66.7% (28.9–100)	p = 0.27	100% (100–100) 91.7% (76–100)	p = 0.34
**Location**Pelvis (N = 11)Other (N = 9)	60.0% (17.1–100)66.7% (13.3–100)	p = 0.87	100% (100–100) 87.5% (64.6–100)	p = 0.69
**Tumor size**<8 cm (N = 10)≥8 cm (N = 10)	100% (100–100) 50% (1–99)	**p = 0.04/p = 0.02**	100% (100–100) 90% (71.4–100)	p = 0.11/NS
**Surgical Margin**Negative (N = 14)Positive (N = 6)	75% (32.5–100)50% (1–99)	p = 0.38	92.3% (77.5–100) 100% (100–100)	p = 0.15/NS
**Adjuvant treatment**Yes (N = 3)No (N = 17)	50.0% (0–100) 66.7% (28.9–100)	p = 0.51	100% (100–100) 93.3% (80.7–100)	p = 0.95
**Neoadjuvant treatment**Yes (N = 6)No (N = 14)	100% (100–100) 71.43% (38–100)	p = 0.13/NS	100% (100–100) 91.67% (76–100)	p = 0.42
**Reintervention**Yes (N = 12)No (N = 8)	66.7% (13.7–100)60.0% (17.0–100)	p = 0.72	85.7% (59.8–100) 100% (100–100)	**p = 0.05/p = 0.026**

## Discussion

Limb-salvage techniques have become the standard of care for managing MBTs, with amputation reserved for cases where limb preservation is not feasible or would cause unacceptable functional loss, such as after major nerve resection or incomplete tumor excision. Limb-sparing reconstruction options include allografts, endoprostheses, and recycled autografts, among others [4], [19]. The use of AR offers several advantages. Because the graft is derived from the patient’s own irradiated bone, it provides an exact size and shape match, is cost-effective, and eliminates the need for allograft bone storage. Moreover, AR using ECI is a valuable alternative when other reconstructive options are limited, such as in cases of allograft unavailability or concerns about viral transmission from cadaveric grafts [8], [10], [20]. However, this technique is contraindicated in the presence of pathological fractures or when the tumor-bearing bone is structurally compromised [20]. When complete tumor resection is achievable, limb-sparing surgery optimizes functional outcomes while maintaining adequate LC. [10].

In our series, the 1-year LC rate was 100%, decreasing to 62.5% at 2 years. Despite the limited cohort size, these results suggest LC rates comparable to those reported in the literature, when taking account the large proportion of large tumors [21]. According to published reports, local recurrences were rarely reported [6], [7], [10], [22]. In our study, 3 local recurrences were observed among 20 patients. This relatively high rate could probably be explained by the important tumor size (>8cm) in half of the patients in the cohort, and frequent pelvic location. Two of these occurred in patients with R1 resection margins, which may have contributed to tumor relapses. These patients developed local relapse in close proximity to the positive resection margins. This finding underscores the importance of achieving wide resection margins during en-bloc resection, to maintain optimal LC [23]. The patient, who had experienced a local recurrence despite an R0 resection, developed a recurrence in the form of muscle invasion near the surgical site.

A major limitation of this technique lies in the assessment of surgical margins. This challenge stems from the need for the surgeon to dissect (“peel”) the surrounding soft tissues from the resected bone specimen. These tissues are subsequently marked, and submitted separately for histopathological evaluation, whereas the tumor-bearing bone is typically transferred intact to the radio-oncology department. In some cases, the thinness of the surrounding soft tissues, sometimes just a fascia or a few millimeter thick muscle layer, may be extremely difficult to process for the surgeon, and to analyse for the pathologist, especially in complex locations, typically the pelvis or shoulder girdle. Nevertheless, in sarcomas with soft tissue extension, the extra-osseous component generally represents the critical area for achieving wide surgical margins. Surgeons therefore tend to apply heightened caution in these regions, which may, in turn, enhance the reliability of subsequent histopathological margin assessment.

In our study, each resected tumor-bearing bone received a single fraction of 50 Gy. This single-fraction irradiation represents a massive single dose compared with the standard fractionated external-beam radiotherapy used for malignant bone tumors and is intended to achieve complete tumor cell eradication [6]. During irradiation, the bone was placed in a water-filled canister to ensure uniform dose distribution, exposing all tumor cells to the same radiation fraction and eliminating regions of underdosing [10]. Administering more than 50 Gy per fraction is unlikely to provide additional benefit, as it would prolong treatment time and may fragilize the resected bone [6].

The most commonly reported complications after ECI and reimplantation are wound-related, such as infections, and mechanical complications, including delayed union [9], [10], [24]. In our cohort, 14 of 20 patients experienced postoperative complications, most of which were orthopedic (N = 6, 30%) including prosthetic dislocation, implant loosening, and hardware-related discomfort. Neurological pain was reported in 4 patients (N = 4, 20%) and 4 other patients experienced wound dehiscence, infection, secondary hip osteoarthritis and pressure sores. Moreover, in this selected cohort of patients with unfavorable tumor configuration (55% of pelvic tumors) non orthopedic complications could be related to surgery itself rather than to the AR procedure.

Functional outcomes were evaluated using a modified Mankin functional scale [7], [13]. In our series, 12 patients (60%) achieved excellent or good outcomes, 4 (20%) had fair results, and 4 (20%) were classified as poor. These results are lower than those reported in other studies, which show 70–90% excellent to good outcomes [6], [25], [26]. The mechanical value of a recycled autograft bone may be questioned, and data regarding loss of resistance and strength of AR are lacking. However, no mechanical failure – related to the AR process- was to deplore, even though high constrain bone segments were reconstructed. Stiff orthopedic fixation and the addition of vascularized fibula graft in weightbearing lower extremity segmental reconstructions (femur and tibia) and the filling of the reimplanted bone with cement, especially in the pelvis, may be used to enhance mechanical resistance. The use of antibiotic-loaded cement, with doses of antibiotics comparable to the cement spacer used in the treatment of orthopaedic infections, has an additional protective efect againt infection. Hence, even though some cases of infections were deplored in our series, the rate remained comparable to the literature In this cohort, two significant prognostic factors were identified. Tumors ≥ 8 cm were significantly associated with a poorer LC, and surgical reinterventions were associated with a reduced OS. Advanced age, larger tumor size, and high-grade sarcoma have previously been reported as negative prognostic factors in MBTs, further studies are needed in order to confirm our new findings [27].

Another limitation of the study is the small and heterogeneous cohort, encompassing a wide range of histological subtypes, grades, stages, anatomical locations, reconstruction techniques, and (neo)adjuvant treatment modalities.

However, this heterogeneity also underscores the broad applicability of AR across diverse clinical scenarios. In contrast, more commonly employed reconstructive strategies, particularly tumor endoprostheses and allograft reconstruction—may be restricted by limited availability or anatomical constraints in certain locations.

Although 3D-printed implants are emerging as an increasingly utilized alternative, their use remains constrained in some settings due to issues related to availability, manufacturing time, and higher associated costs.

In conclusion, AR using ECI represents a promising treatment alternative for MBTs, demonstrating favorable functional and oncological outcomes, in appropriately selected patients.

## Declaration of Competing Interest

The authors declare that they have no known competing financial interests or personal relationships that could have appeared to influence the work reported in this paper.

## References

[1] Jl F, Sp T. Bone Cancer: Diagnosis and Treatment Principles. Am Fam Physician [Internet]. Am Fam Physician; 2018 [cited 2025 Feb 20];98(4). Available from: https://pubmed.ncbi.nlm.nih.gov/30215968/. 30215968

[2] Franchi A. (2012). Epidemiology and classification of bone tumors. Clin Cases Miner Bone Metab off J Ital Soc Osteoporos Miner Metab Skelet Dis.

[3] Sbaraglia M., Bellan E., Dei Tos A.P. (2020). The 2020 WHO Classification of Soft Tissue Tumours: news and perspectives. Pathologica.

[4] Haynes K.K., Rosenthal H.G. (2020). The Ever-changing World of Limb Salvage Surgery for Malignant Bone Tumors. Nurs Clin North Am.

[5] Spira E., Lubin E. (1968). Extracorporeal irradiation of bone tumors. a preliminary report. Isr J Med Sci.

[6] Davidson A.W., Hong A., McCarthy S.W., Stalley P.D. (2005). En-bloc resection, extracorporeal irradiation, and re-implantation in limb salvage for bony malignancies. J Bone Joint Surg Br.

[7] Hong A., Stevens G., Stalley P., Pendlebury S., Ahern V., Ralston A. (2001). Extracorporeal irradiation for malignant bone tumors. Int J Radiat Oncol Biol Phys.

[8] Böhm P., Fritz J., Thiede S., Budach W. (2003). Reimplantation of extracorporeal irradiated bone segments in musculoskeletal tumor surgery: clinical experience in eight patients and review of the literature. Langenbecks Arch Surg.

[9] Jones C.W., Shatrov J., Jagiello J.M., Millington S., Hong A., Boyle R. (2017; 99-B(12):1681–8.). Clinical, functional and radiological outcomes of extracorporeal irradiation in limb salvage surgery for bone tumours. Bone Jt J.

[10] Hong A.M., Millington S., Ahern V., McCowage G., Boyle R., Tattersall M. (2013). Limb preservation surgery with extracorporeal irradiation in the management of malignant bone tumor: the oncological outcomes of 101 patients. Ann Oncol off J Eur Soc Med Oncol.

[11] Oken M.M., Creech R.H., Tormey D.C., Horton J., Davis T.E., McFadden E.T. (1982). Toxicity and response criteria of the Eastern Cooperative Oncology Group. Am J Clin Oncol.

[12] https://www.youtube.com/watch?v=vNTg5Neo2HE.

[13] Mankin H.J., Gebhardt M.C., Jennings L.C., Springfield D.S., Tomford W.W. (1996). Long-term results of allograft replacement in the management of bone tumors. Clin Orthop.

[14] National Cancer Institue. National Cancer Institute [Internet]. 2017 Nov 27. Common Terminology Criteria for Adverse Events (CTCAE) [cited 2025 Dec 17]. Available from: https://dctd.cancer.gov/research/ctep-trials/for-sites/adverse-events/ctcae-v5-5x7.pdf.

[15] Goel M.K., Khanna P., Kishore J. (2010). Understanding survival analysis: Kaplan-Meier estimate. Int J Ayurveda Res.

[16] Bland J.M., Altman D.G. (2004). The logrank test. BMJ.

[17] Anderton J., Moroz V., Marec-Bérard P., Gaspar N., Laurence V., Martín-Broto J. (2020). International randomised controlled trial for the treatment of newly diagnosed EWING sarcoma family of tumours - EURO EWING 2012 Protocol. Trials.

[18] Whelan J.S., Bielack S.S., Marina N., Smeland S., Jovic G., Hook J.M. (2015). EURAMOS-1, an international randomised study for osteosarcoma: results from pre-randomisation treatment. Ann Oncol off J Eur Soc Med Oncol.

[19] Cirstoiu C., Cretu B., Serban B., Panti Z., Nica M. (2019). Current review of surgical management options for extremity bone sarcomas. EFORT Open Rev.

[20] Puri A., Gulia A., Agarwal M., Jambhekar N., Laskar S. (2010). Extracorporeal irradiated tumor bone: a reconstruction option in diaphyseal Ewing’s sarcomas. Indian J Orthop.

[21] Agarwal S., Rathi A.K., Singh K., Melgandi W., Ansari F.A., Arora S. (2023). Extracorporeal irradiation in malignant bone tumors: Single institution experience and review of literature. J Cancer Res Ther.

[22] Krieg A.H., Lenze U., Schultze L., Gross M.W., Haug M. (2019). Extracorporeal Irradiation and Reimplantation of Tumor-bearing Bone Segments following Diaphyseal Sarcoma Resection at the Tibia. Anticancer Res.

[23] Dickinson I.C., Whitwell D.J., Battistuta D., Thompson B., Strobel N., Duggal A. (2006). Surgical margin and its influence on survival in soft tissue sarcoma. ANZ J Surg.

[24] Kapoor L., Singh H., Sahoo B., Banjara R., Kumar V.S., Bakhshi S. (2023). Factors affecting the incorporation of extracorporeally irradiated autograft for the treatment of bone tumours-a retrospective analysis from a tertiary referral centre. Int Orthop.

[25] Anacak Y., Sabah D., Demirci S., Kamer S. (2007). Intraoperative extracorporeal irradiation and re-implantation of involved bone for the treatment of musculoskeletal tumors. J Exp Clin Cancer Res CR.

[26] Arpornchayanon O., Leerapun T., Pruksakorn D., Panichkul P. (2013). Result of extracorporeal irradiation and re-implantation for malignant bone tumors: a review of 30 patients. Asia Pac J Clin Oncol.

[27] Zhou H., He S., Zhang D., Wang J., Yang X., Jiao J. (2023). Prognostic factors of sarcomas occurring in bone and joint: a SEER based study. Medicine (Baltimore).

